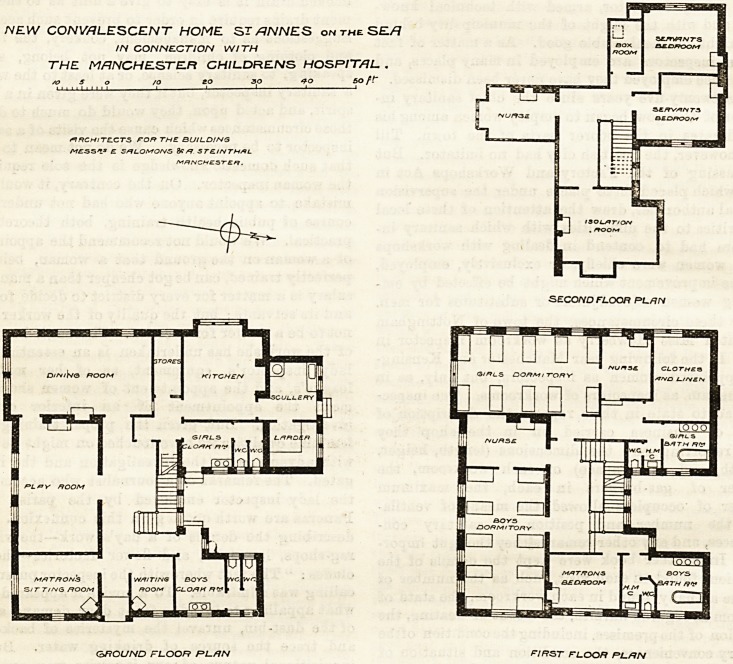# Hospital Construction

**Published:** 1899-02-25

**Authors:** 


### The Institutional Workshop.

### HOSPITAL CONSTRUCTION.

### MANCHESTER CHILDREN'S HOSPITAL—ST. ANNE'S-ON-SEA CONVALESCENT HOME.

The plans of this home, published in The Hospital for January 8th, 1898, show the original design for 24 beds; but the building actually erected accommodates only twenty patients, and some other modifications have been made in order to reduce the cost. The plans now published show that these modifications in no way detract from the excellence of

GROUND FLOOR PLAN

SECOND FLOOR PLAN

FIRST FLOOR PLAN

the general arrangements. On the ground floor the reduction in size has been obtained by omitting a pantry and store-room, and by making one dining-room serve for all the patients; while the girls' cloak-room opens directly on to the grounds, instead of through a small passage. On the first floor the general arrangement is unchanged, but the two dormitories are designed for 10 beds each, instead of 12. The attic plan, not previously published, shows the servants' and nurses' rooms, and the large isolation-room provided in case of need. Standing on a site of three acres, kept, except for its paths, in a "state of nature," so as to be most suitable for a playground, with the sea almost washing its gates, the home must be an enormous boon to the children of Manchester, for which they may well thank Sir W. Agnew's bounty; and, as pointed out before, its plan is a model which deserves careful study.

---

## Figures and Tables

**Figure f1:**